# Inflammatory Bowel Disease Treatment in Cancer Patients—A Comprehensive Review

**DOI:** 10.3390/cancers15123130

**Published:** 2023-06-09

**Authors:** Daniel Conceição, Margarida R. Saraiva, Isadora Rosa, Isabel Claro

**Affiliations:** Department of Gastroenterology, Instituto Português de Oncologia de Lisboa Francisco Gentil, 1099-023 Lisboa, Portugal; dgc1990@gmail.com (D.C.); margarida.rsaraiva@gmail.com (M.R.S.); iclaro@ipolisboa.min-saude.pt (I.C.)

**Keywords:** inflammatory bowel disease, malignancy, immunomodulation, cancer recurrence

## Abstract

**Simple Summary:**

With the increase in life-expectancy and the aging population, patients with inflammatory bowel disease are being exposed to immunomodulating drugs for longer periods. This, as well as the already recognized higher risk of some cancers in these patients when compared to the general population, makes the challenge of managing a patient with inflammatory bowel disease and current or past cancer more common. As large prospective studies are awaited, we summarize the available data on cancer risk in inflammatory bowel disease patients, the risk of cancer recurrence with immunomodulating therapy, the effects of cancer treatment on inflammatory bowel disease, and current recommendations on how to balance the risks and benefits of different treatments in these patients.

**Abstract:**

Inflammatory bowel disease (IBD) is a chronic disease for which medical treatment with immunomodulating drugs is increasingly used earlier to prevent disability. Additionally, cancer occurrence in IBD patients is increased for several reasons, either IBD-related or therapy-associated. Doctors are therefore facing the challenge of managing patients with IBD and a past or current malignancy and the need to balance the risk of cancer recurrence associated with immunosuppressive drugs with the potential worsening of IBD activity if they are withdrawn. This review aims to explore the features of different subtypes of cancer occurring in IBD patients to present current evidence on malignancy recurrence risk associated with IBD medical therapy along with the effects of cancer treatment in IBD and finally to discuss current recommendations on the management of these patients. Due to sparse data, a case-by-case multidisciplinary discussion is advised, including inputs from the gastroenterologist, oncologist, and patient.

## 1. Introduction

Crohn’s disease (CD) and ulcerative colitis (UC) are chronic idiopathic inflammatory bowel diseases commonly diagnosed in early adulthood [1]. It is estimated that at least 0.4% of the Western world’s population lives with inflammatory bowel disease (IBD), with a calculated total of 4.9 million cases worldwide in 2019 [2,3]. The prevalence of IBD is globally increasing, meaning it may become a major public health burden, with high disability-adjusted life years [3].

According to population studies, IBD patients do not show an increase in the overall risk of cancer when matched with the general population [1].

Nowadays, the global prevalence of cancer is increasing, largely as the average life expectancy is rising. It is expected that over one third of the population in developed countries will be diagnosed with cancer during their lifetime [4,5].

Therefore, most cancers found in IBD patients will be sporadic, and gastroenterologists will be increasingly managing patients with IBD and current or past cancers. Regardless of the cancers’ etiology, the therapeutic management of IBD may have to be adjusted. Although oncological treatment must be prioritized, the need to maintain IBD control makes the management of these patients challenging, and this will be the focus of the current review.

## 2. Inflammatory Bowel Disease and Malignancies

When an IBD patient is diagnosed with a malignancy, although it will most likely be a sporadic cancer, IBD-associated cancers or a drug-related etiology must also be remembered. This is why IBD patients should be encouraged to engage in national screening programs, as well as in IBD-specific endoscopic surveillance for colorectal cancer and in surveillance for cholangiocarcinoma when primary sclerosing cholangitis is also diagnosed [1,6].

In this section, we will discuss the cancer subtypes that are more frequently found in subsets of IBD patients, such as colorectal cancer (CRC) in colonic IBD, anal and small bowel cancer in perianal/small bowel Crohn’s disease, cholangiocarcinoma in IBD with primary sclerosing cholangitis (PSC), and hematological malignancies.

We will further discuss the malignancy risks associated with therapy, namely, regarding thiopurines, antitumor necrosis factor α (anti-TNFα) in monotherapy or in association with immunomodulators, anti-integrins, anti-IL-12/23, and Janus kinase (JAK) inhibitors.

### 2.1. IBD Related Cancers

**Figure 1:**
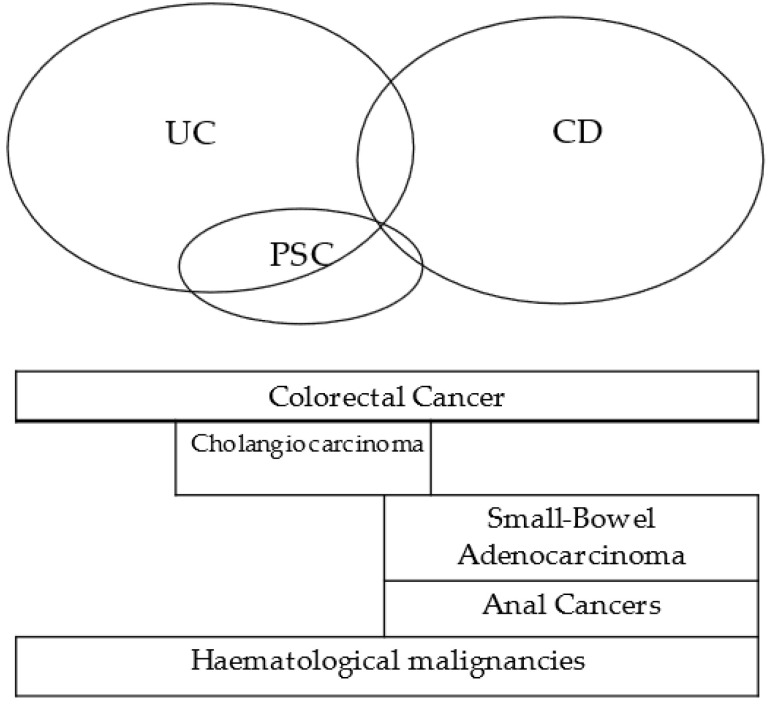
IBD related cancers.

Increased risk of cancer in inflammatory bowel disease (IBD) patients has been mostly attributed to long-standing chronic inflammation, with the contribution of genetic alterations and environmental factors. Oxidative stress and several cytokines that increase with inflammation may be able to induce both genetic and epigenetic DNA changes, possibly explaining the known link between inflammation and cancer [6]—Figure 1.

#### 2.1.1. Colorectal Cancer

Both sporadic CRC and colitis-associated CRC may develop in IBD patients [6]. A CRC increased risk, at least 2-fold higher than in the general population, in patients with ulcerative colitis or with colonic CD is well established [7,8,9,10,11,12].

Over the past 20 years, studies have shown a decline in IBD-associated CRC rates [7,8,9,10]. This may be attributed to better inflammation control with newer drugs and strategies, implementation of colonoscopic surveillance programs, increased surgical (colectomy) approach in some countries, and the possible chemopreventive effect of 5-aminosalicylates (5-ASAs) [6,11,12].

Even though colitis-associated cancer shares many features with sporadic colorectal cancer, there are some differences, especially concerning the timing and frequency of the alterations in the dysplasia–carcinoma sequence [13]. Chronic inflammation plays an important role, and it is believed to be the underlying cause of colitis-associated cancer, with oxidative-stress-induced DNA damage resulting in the activation of procarcinogenic genes and the silencing of tumor-suppressor pathways. This leads to a field defect that explains the increased rates of both synchronous and metachronous neoplastic lesions.

Only up to 15% of all colorectal cancers that occur in IBD are diagnosed within the first 7 years of disease, with a linear increase in CRC occurrence beyond that. For this reason, most societies agree on starting endoscopic surveillance after 8 years of disease [10]. An exception may be made when IBD is diagnosed after 45—these patients may explain the 15% of CRCs diagnosed in IBD before 8 years of disease duration in some studies, and, in them, surveillance should probably start at 6 years disease duration or at 50, whichever comes first [14,15].

The individual endoscopic findings (colonic strictures, presence of dysplastic lesions, colonic extension of the disease, grade of activity) as well as family history of CRC (especially if below 50) and associated primary sclerosing cholangitis should be taken into account to set the surveillance program (Table 1).

Currently, most international guidelines favor surveillance by total colonoscopy with high-definition white light with/or without chromoendoscopy (virtual or conventional), with targeted biopsies. Dye-spray chromoendoscopy must be used when standard definition colonoscopes are used and random biopsies must be added in cases of deficient bowel preparation, active inflammation, or high-risk patients (PSC, tubular colon, previous dysplasia) [16]. This should ideally be done while patients are in disease remission [17,18]. During surveillance, any dysplastic lesion found should be endoscopically removed. In cases not amenable to endoscopic resection, as well as in cases of high-grade invisible or multifocal low-grade invisible dysplasia, the patient ought to be referred for surgical treatment [19].

#### 2.1.2. Small-Bowel Adenocarcinoma

Small-bowel adenocarcinoma is a rare entity in the general population, but in Crohn’s disease patients, the risk may be 20 to 30 times higher [20].

It typically arises in the distal jejunum or ileum and is more frequent in patients with CD with small bowel involvement for more than 8 years—this subgroup of patients shows an incidence rate of small-bowel adenocarcinoma of 0.5 per 1000 patient years [21]. The finding that small-bowel adenocarcinoma is often associated with previous or synchronous ileal dysplasia is suggestive that it arises from severe chronic ileal inflammation.

Imagological appearance on computed tomography and magnetic resonance (MRI) can be suggestive, but most cases are incidentally diagnosed during surgery [22]. The surgical excision of ileal lesions prevents the risk of subsequent malignant transformation, but no other preventive approach is currently available, besides the potential chemopreventive effect 5-ASA [11].

Moreover, routine surveillance with imaging or endoscopy is not recommended for small-bowel cancers. Alarming features that raise suspicion for this diagnosis include refractory, longstanding structuring disease [19].

#### 2.1.3. Anal Cancers

With an incidence rate of 0.2 per 1000 patient-year, anal cancer in CD patients usually arises in fistulae, including both adenocarcinomas and squamous-cell carcinomas and showing no consistent relationship with HPV infection [23,24].

Long-standing (>10 years) anal or perianal CD is associated with an increased risk of anal cancer, and chronically active fistulizing disease may lead to an advanced cancer stage at diagnosis [24]. Due to a low absolute risk, there is no recommendation for a formal surveillance program. However, especially in long-standing perianal CD, any changes in anal symptoms should lead to further evaluation, namely an MRI and/or biopsy of suspicious lesions or the curettage of fistula tracts [19].

#### 2.1.4. Cholangiocarcinoma

The risk of cholangiocarcinoma in patients with IBD is approximately 2 to 4 times higher than in the general population, and when associated with PSC the risk is 400 times higher than in the general population [19]. Population-based studies found that 27–37% of incident cholangiocarcinomas are detected within 1 year of PSC diagnosis [25].

Due to the prognostic implications of cholangiocarcinoma, bi-annual or annual surveillance with imaging is generally advised. Most experts recommend noninvasive annual imaging of the biliary tract (magnetic resonance cholangiopancreatography or ultrasonography) and additional serum carbohydrate antigen (CA 19-9) measurement [6,19].

The lifesaving treatment for cholangiocarcinoma is orthotopic liver transplantation. However, PSC reoccurs in approximately 20–25% of patients within 10 years after transplantation [19]. Despite this approach, the prognosis of cholangiocarcinoma remains poor [6].

#### 2.1.5. Hematological Malignancies

Several studies point to an increased risk of hematological malignancy in IBD, and a systematic review and meta-analysis has confirmed this; however, this work included patients who received immunosuppressive therapy [26]. Therefore, currently there is insufficient evidence to determine whether the risk is independent of the concomitant effect of therapy or other risk factors [19].

### 2.2. IBD Drug-Related Malignancies

The exact cancer risk associated with IBD therapy is difficult to access due to overlap of the IBD risk itself [19].

Table 2 summarizes the cancer risk associated with the use of medical therapies in IBD.

#### 2.2.1. Thiopurines

Thiopurines act through direct interference with DNA replication [27,28,29], which both explains their immunomodulating effect and their associated cancer risk.

Thiopurines’ risk of Epstein–Barr virus (EBV)-associated lymphoma is unpreventable during use, strongly increases with age, and is doubled in men, but it is reversible on drug withdrawal [27,28,29]. This risk gains more importance in male patients over 65 years old, who may also be at increased risk of urinary tract cancers (especially if smokers) [28,29]. For this reason, many authors consider it wise to limit the use of thiopurines to patients younger than 65 years [1].

Besides that, EBV-seronegative patients, particularly young men, are at risk of fatal macrophagic activation syndrome and post-mononucleosis lymphoma after EBV-primary infection under exposure to thiopurines [27]. In this group of patients, the use of thiopurines is generally reserved for severe uncontrolled IBD with no alternative therapy.

Limited data show a possible increase in other hematological malignancies, namely acute myeloid leukemia and myelodysplastic syndromes [19,28].

Regarding solid cancers, the increase in non-melanoma skin cancer [NMSC] risk in patients with IBD exposed to thiopurines has been conclusively shown by several systematic reviews and meta-analyses, and the total cumulative dose may have an impact [29,30,31]. Skin cancer screening and sun protective measures are both recommended.

The available literature on thiopurines associated risk of cervical high-grade dysplasia and cancer is controversial [32], and women undergoing thiopurines therapy are encouraged to receive anti-HPV vaccination and to participate in screening programs available for the general population [32,33,34].

#### 2.2.2. Anti-TNFα

Anti-TNFs are biologic drugs that bind directly to one of the major pro-inflammatory cytokines in IBD, TNFα, blocking its action.

There is no evidence of an overall increased risk of cancer in IBD patients treated with anti-TNFα monotherapy, although the risk of lymphoma and melanoma may be increased [19,27]. Available studies may be limited by confounding factors such as prior or concomitant thiopurine exposure, and the increase in risk, if real, will probably be very small. Although there is insufficient data to recommend specific screening, sun protection and skin cancer surveillance should be promoted [1,19].

#### 2.2.3. Anti-TNFα and Immunomodulator

Anti-TNFα and thiopurines combination therapy seems to be associated with a higher lymphoma risk than thiopurines or anti-TNFα monotherapy. Studies suggest that the risk of lymphoma is increased twofold in patients exposed to anti-TNFs alone and up to sixfold in those exposed to combination therapy with thiopurines [27].

Knowing that elderly patients have an increased lymphoma risk, combination therapy should be used sparingly in this age group. Particular attention should also be given to male CD patients below 30 years of age, in whom the thiopurine plus anti TNF combination has been shown to increase the risk for hepatosplenic T-cell lymphoma—a very rare and aggressive cancer—if used for more than 2 years [27].

Data on the risk of lymphoma in IBD patients exposed to anti-TNFα in combination with methotrexate are insufficient [19].

#### 2.2.4. Anti-Integrins

Vedolizumab is a biologic drug that blocks T-lymphocyte migration into the gut, acting on an integrin that is mostly expressed in the gastrointestinal tract, which confers the molecule its specificity.

Available evidence derives from short- and medium-term studies that do not show an increased risk of malignancy in patients with IBD treated with vedolizumab (VDZ) [35]. However, more studies are needed to draw a definite conclusion.

#### 2.2.5. Anti IL-12/IL-23

Ustekinumab targets a subunit common to both IL-12 and IL-23, blocking their action and therefore modulating lymphocyte function.

Short- and medium-term evidence does not show an increased risk of malignancy in patients with IBD treated with ustekinumab (UST). Data from a long-term registry of psoriatic patients treated with UST revealed a similar cancer risk than the general population, although the population and the drug dosage are both different [36,37].

#### 2.2.6. JAK Inhibitors

Tofacitinib was the first small molecule from this new group to be approved in IBD [38]. Janus kinases (JAK) are enzymes involved in intracellular signaling, namely in the inflammatory response pathway.

There is no evidence that the overall risk of cancer is increased in IBD patients treated with JAK inhibitors, although long-term evidence is lacking [19].

The available data from rheumatoid arthritis (RA) patients under tofacitinib are, however, more concerning, with some studies showing a greater incidence risk of malignancies, namely lymphoma and lung cancer, especially in older patients with tobacco exposure [39]. Although these results are nonconsensual, presently these drugs should be avoided in any IBD patient with a current or recent cancer since several other therapies with accumulated safety evidence are available.

#### 2.2.7. Methotrexate

Methotrexate diminishes immune responses by several mechanisms, including interfering with DNA synthesis.

Data on methotrexate use in IBD and its associated risk of cancer are limited. Methotrexate is mostly used in rheumatologic patients, while thiopurines are predominantly the classic immunomodulators of choice in IBD.

Methotrexate-treated patients may be at increased risk of NMSC—in one of the first studies that reported this positive association, the number of events was low [31]; a more recent Danish case–control study reports a dose-dependent increase in risk for cutaneous squamous cell carcinoma and basal cell carcinoma [40]. Nevertheless, there is still insufficient evidence to support specific screening for skin cancer.

#### 2.2.8. Novel IBD Drugs

Risankizumab, an IL-23 inhibitor, has shown good treatment outcomes for CD [41]. The leukocyte migrating inhibitor sphingosine 1-phosphate (S1P) receptor modulator, ozanimod, is already used for UC treatment in the United States [42]. A novel JAK inhibitor, named upadacitinib, has already revealed satisfactory results both in UC and CD treatment [43]. Since these have only recently been approved for clinical practice, long-term security data are needed before considering its use in patients in the oncology setting.

## 3. Managing Inflammatory Bowel Disease during Oncological Treatment

As IBD treatment approaches evolve, prioritizing an early and diverse use of immunomodulating (IM) drugs such as thiopurines or biological molecules, as well as adopting more ambitious targets for IBD treatment [38], patients are more frequently exposed to these agents and for a longer period of their lives. On the other hand, with the aging population and longer life expectancy, elderly IBD patients become more prevalent [44], and cancers’ incidence increases [45]. Hence, gastroenterologists and oncologists are more frequently facing IBD patients with a past or present cancer diagnosis, commonly under immunomodulation.

There is a lack of evidence about the best way to manage these patients. First of all, most clinical trials on IBD exclude patients with past malignancies. Additionally, guidelines traditionally recommend IBD patients with a previous cancer to avoid IM for 2–5 years [4], and clinicians, out of concern that these drugs might increase the risk of recurrence of the previous cancer or of the development of a new primary, avoid its use in this group of patients [46]. As new molecules join the therapeutical options for IBD treatment, the knowledge gap increases. All of these contribute to the paucity of evidence in this area, thus making the management of these patients challenging.

When dealing with a new cancer diagnosis, multidisciplinary management, including an oncologist and a gastroenterologist, and a personalized case-by-case approach are advised. First, the malignancy characteristics should be discussed, namely its location, histology, staging, prognosis, and therapeutic plan—its life-threatening character makes this assessment the priority [47]. Second, IBD past history and severity should be analyzed, medical IBD treatment potential effects on the progression/recurrence of cancer, and also the influence of the cancer treatment on IBD should be discussed [4]. After all these considerations, the multidisciplinary team may finally balance the risk of IBD exacerbation when IBD medication is stopped with the risks of maintaining it for the cancers’ evolution (progression/relapse/recurrence) [48].

Another factor to consider when reflecting on changing IBD treatment is that little is known about the potential impact of the malignant disease on IBD evolution. The current data are scarce, but a prospective study showed that, although the diagnosis of a non-intestinal cancer led to an important change in medical IBD treatment drugs (lesser use of thiopurines and anti-TNFα), it did not interfere with IBD inflammatory activity, with a long-term course equal to IBD patients without cancer [49].

### 3.1. Risk of Maintaining IBD Immunomodulator Treatment

The main concern about maintaining/starting IM drugs in present/past cancer patients with IBD is the risk of recurrence or new primary cancer.

This is even more important considering that patients with a past history of cured cancer still maintain a 14% higher risk of developing a second malignancy than the general population [50], which may be justified by both a higher genetic susceptibility, specific exposures, and also an eventual contribution from the prooncogenic features of some cancer therapy. The already mentioned increased risk of cancer known to exist in IBD patients adds to this. In fact, according to ECCO guidelines, IBD patients with previous cancer have a 2-fold increased risk of developing a new one or experiencing a recurrence [4].

However, there may also be an increased recurrence risk intrinsic to IM drugs.

Pen [51] was the first to describe the risk of using immunosuppression in post-kidney transplant patients with previous cancer. In fact, when analyzing cancer recurrence after transplantation in 939 patients with previous cancer treatment, recurrence rates as high as 22% were found and, most importantly, with different recurrence rates for different types of cancers, which allowed a classification of the tumors in accordance with their low, intermediate, or high risk of recurrence following immunosuppression, and this classification was used to guide medical decisions until recently. In this cohort, the risk of cancer was significantly higher for patients that initiated immunosuppression within 2 years of cancer treatment (53%) when compared to 2–5 years (34%) or more than 5 years (13%). However, there are several major limitations to this study—it was a retrospective study, with a possible referral bias; there is no control population (and colon cancer, for instance, was classified as intermediate risk due to a recurrent rate that is similar to the one found in the general population, with no IS); and the recurrence risk was not stratified for the type of immunosuppression.

There are currently much more robust data from the IBD population, and these should be used for decision making, replacing historical data.

The Cancers Et Surrisque Associé aux Maladies inflammatoires intestinales en France (CESAME) study was one the first scientifically robust studies in this field. This prospective multicenter study included 405 IBD patients with previous cancer and confirmed an increased risk of developing an incident cancer (with predominance of new cancers rather than recurrences). However, although patients exposed to IM (thiopurines) showed higher rates of new/recurrent cancer when compared to patients without IM, the difference did not reach statistical significance, leading to the conclusion that IM treatment was not associated with an increased risk of incident cancer [52].

Since these IBD patients were mostly taking thiopurines (the number of patients on anti-TNFα or methotrexate being too small for a proper risk assessment), there may be a bias from excluding patients with previous history of lymphoma or NMSCs from IM, and a careful interpretation of these results is advised.

Other studies further confirmed CESAME findings and included results from anti-TNFα drugs. A retrospective multicentric study by Axelrad et al. including 333 IBD patients with a history of cancer showed no difference in cancer recurrence or new cancer occurrence between patients exposed to thiopurines, methotrexate, anti-TNFα, or combination therapy, not even when these patients were compared to those not exposed to IM. Additionally, the timing of IM exposure after cancer diagnosis did not interfere with the risk of incident cancer [53].

In a Danish case–cohort study that included patients with immune-mediated disease (IBD, RA, or psoriasis) and cancer, anti-TNFα treatment was not associated with an increase in new or recurrent cancer when compared to the control group, and the timing (before or after 2 years from cancer diagnosis) of anti-TNFα therapy was also unrelated to a higher recurrence risk. Another American prospective multicenter study of 6273 patients with CD showed that cancer recurrence rates in patients taking anti-TNFα were similar to the general population [54].

A promising longitudinal study, the SAPPHIRE registry, is an ongoing prospective study that intends to determine the risk of cancer recurrence in IBD patients with a previous cancer and exposure to IM. In 2020, it had included 170 patients with IBD and a history of a first cancer within the last 5 years, and data revealed that exposure to thiopurines or biologic drugs was not associated with an increased cancer risk, although combination therapy was. As more patients enroll and with a longer follow-up period, further elucidating data are expected [55].

A similar study in Europe, the I-CARE (IBD Cancer and Serious Infections in Europe), is currently also in the follow-up stage.

In a systematic review and meta-analysis [56] including 16 studies and a total of 11,702 IBD, rheumatoid arthritis (RA), and psoriasis patients with previous malignancy, cancer recurrence rates were the same for patients receiving no IM, treated with anti-TNFα, with thiopurine, or with combination therapy. A numerically higher recurrent cancer risk was found in patients undergoing combined immunosuppression, but this had no statistical significance. As for the timing for IM initiation, no conclusions can be made because the median interval in these studies was 6 years.

These data have been extensively corroborated by various studies on RA patients with a prior cancer that were treated with anti-TNFα drugs vs. conventional disease-modifying antirheumatic drugs (DMARDs). For instance, Dixon et al. analyzed data from the British Society for Rheumatology Biologics Register and showed no difference in the development of new or recurrent cancer between RA patients treated with anti-TNFα vs. DMARDs and even a lower rate of malignancy in the first group [57]. The same was concluded by a prospective study based on data from the German Biologics Register [58].

Besides anti-TNFα, other novel biologic molecules have been more recently approved as IM drugs in IBD. Some data on VDZ and UST and their safety in cancer patients are already available.

A retrospective cohort study included 390 patients with IBD and a previous history of cancer exposed either to VDZ (37), UST (14), anti-TNFα (41), or conventional IM (31) and 267 patients not exposed to immunomodulation following cancer diagnosis. After a median follow-up period of 52 months, 20% of all patients under IM developed an incident cancer, but exposure to VDZ or UST did not increase the risk of new or recurrent cancer when compared with exposure to conventional IM, anti-TNFα, or no immunosuppression, not even when analyzing only patients exposed to biologic treatment within 5 years of cancer diagnosis (median time interval was 2 years for VDZ and only 5 months for UST) [59]. A similar study that included 96 IBD patients with a prior malignancy on VDZ treatment showed that treatment with VDZ did not increase incident cancer when compared to control patients [60].

Despite these results, the small number of IBD patients in each drug group should be taken into consideration, as well as the intrinsic heterogeneity of the included cancer types, location, histology, and even cancer treatment, as well as the short follow-up times.

Based on the available data, a careful approach was recommended in the latest update on the Guidelines on Inflammatory Bowel Disease and Malignancies issued by ECCO [19]. Their main recommendations are reviewed below:

Patients with active cancer should preferably discontinue thiopurines, with the exception of patients with non-aggressive basal cell carcinoma (BCC) or preneoplastic lesions after successful endoscopic or surgical removal (such as non-aggressive BCC, pre-neoplastic lesions of the cervix, or sporadic colonic polyps), which may continue thiopurines therapy keeping a close surveillance.

Data are insufficient to make recommendations regarding the safety of the use of methotrexate in IBD patients with prior malignancies.

Anti-TNFα drugs may be used in patients with IBD and current or previous cancer, but data on individual cancer types and the timing of treatment with anti-TNFα are lacking.

For more recent drugs, such as VDZ, UST, and JAK inhibitors, data are limited. VDZ and UST do not appear to increase the risk of new or recurrent cancer. Evidence on JAK inhibitors is insufficient.

### 3.2. Recommendations for Management in Clinical Practice

Due to the small amount of evidence available, managing these patients is challenging and should be faced by a multidisciplinary team.

**Figure 2:**
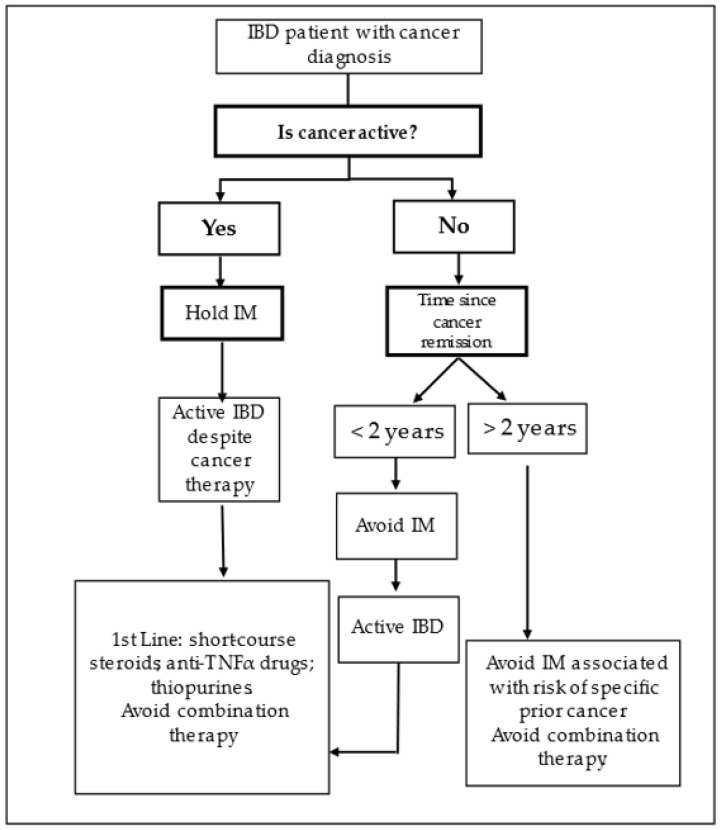
IBD management algorithm in cancer patients.

The most concordant approach still relies on risk stratification, both for cancer and for IBD, since both may affect patients’ outcomes and quality of life (Figure 2).

The updated version of ECCO Guidelines on Malignancies makes no reference to the poor evidence-based cut-off periods of 2 and 5 years and leaves the management of IM in these patients to a multidisciplinary decision [19]—hopefully this will be the future tendency in clinical practice as more and more robust studies on the safety of current IM drugs emerge.

Some of the most consensual options are to try to avoid drugs associated with an increased risk of the cancer previously diagnosed on the patient (thiopurines in lymphoma patients, for instance) and, if possible, to follow the measures below (Table 3):

### 3.3. Cancer Treatment Effect on IBD

On the other hand, some consideration must be given to cancer’s treatment effect on IBD course, for which only limited evidence is available [61,62].

A systematic review including a total of 1298 patients with IBD who received cancer treatment revealed that, despite the fact that the majority of IBD patients maintained IBD remission during and after cancer treatment, the overall occurrence of IBD flares after cancer treatment still accounted for 30%, although the majority of these were manageable and did not interfere with cancer treatment [61]. A French case–control study showed that IBD patients had similar percentages of years with active disease before and after cancer diagnosis and also that it was not significantly different from IBD patients without cancer history [49].

Axelrad [62], in a cohort that included 84 IBD patients with an extraintestinal solid malignancy, tried to assess cancer’s treatment effect on IBD course. The main findings were that 17.4% of patients experienced a flare within 5 years after cancer treatment and that more patients achieved sustained remission while undergoing cytotoxic chemotherapy than those receiving hormonal therapy (with or without associated cytotoxic treatment), suggesting that cytotoxic chemotherapy may induce remission in IBD patients and that hormonal therapies for cancer may increase the risk of an IBD flare. Another prospective study from the same author looking at IBD patients under hormonotherapy for breast/prostate cancers revealed that hormone monotherapy and combination cytotoxic and hormone therapy are associated with IBD relapses [63].

However, studies with larger sample sizes are needed in order to further understand the real effect of chemotherapy on IBD. Only then may specific patient-tailored chemotherapy regimens for IBD patients be designed.

Until then, it is important to remember in clinical practice that cancer treatment should be prioritized in IBD patients. This gains special relevance after a retrospective unicenter study showed that patients with IBD and CRC experience more changes in their cancer treatment compared with patients without IBD, mostly due to delays in their cancer treatment [64].

When administering chemotherapy in IBD patients, caution must be applied when interpreting the occurrence of chemotherapy-related side effects such as diarrhea (for example, with 5-fluorouracil and irinotecan [65]) because they can mimic an IBD flare [66], and infectious aetiologies should also be excluded.

Similarly, another differential diagnosis with an IBD flare is checkpoint-inhibitor-induced colitis. This is a dose-dependent immune-related adverse effect that usually improves with steroids. If not, alternative therapy is anti-TNFα [67]. This is especially relevant since evidence suggests that gastrointestinal toxicity secondary to immune check point inhibitor treatment may be increased in patients with IBD compared to non-IBD patients [61].

Concerning radiotherapy in IBD patients, there are limited data, and most oncologists avoid pelvic radiotherapy in IBD patients due to a historically described higher risk of toxicities and flares [68]. Additionally, according to the National Comprehensive Cancer Network (NCCN) guidelines [69], active/inactive inflammatory diseases of the rectum are absolute/relative contraindications to external beam radiation therapy for prostate cancer.

However, a retrospective study from Mount Sinai Hospital reported an unaltered 5-year survival rate of IBD patients with rectal cancer treated with radiation therapy and no increased gastrointestinal toxicity [70]. Besides that, a systematic review showed its safety for use on IBD patients, with acceptable toxicity profiles, with a similar occurrence of flares between patients treated with radiotherapy or surgery [71], supporting that IBD should not be considered a contraindication for radiation, as reviewed by Bodofsky et al. [72].

## 4. Conclusions and Future Directions

Currently, gastroenterologists and oncologists are dealing with more IBD patients with a cancer diagnosis, either IBD-related, sporadic, or iatrogenic. Due to the lack of substantial clinical data on the management of IBD after a diagnosis of cancer, this reality is a true challenge.

Recommendations were mainly based on data from transplantation patients or other auto-immune diseases, and most guidelines and expert opinions recommended stopping IM during active cancer treatment and only resuming it after 2 to 5 years, avoiding combinations of IM drugs [4].

However, as more recent data are collected, they show that risk for recurrent and new cancer in patients with IBD is not increased by thiopurines or anti-TNFα agents [53], and possibly not even by new biologics such as VDZ and UST [59].

Although available studies have some limitations, clinical practice must begin to change, as has already been proposed by ECCO [19].

Hopefully, data from ongoing studies such as the SAPPHIRE registry or the I-CARE may elucidate on this matter and further help to stratify patents’ risks.

Additional studies are also lacking in terms of IM safety on in situ carcinomas or premalignant conditions, such as Barrett Esophagus. This matter should also be addressed in the future in order to guide IBD patients’ management [73].

Until further long-term, prospective studies are available, it is advised to carefully approach these patients, always on a case-by-case basis and in multidisciplinary approaches. Factors to consider are cancer type and prognosis, cancer risk of recurrence, cancer treatment, and the previous behavior of IBD as well as its severity. The patient should also take part in the making of these decisions.

Last but not least, all IBD patients, but especially those after a cancer diagnosis, should adopt a healthy lifestyle and be enrolled in general population and in IBD-specific screening programs.

## Figures and Tables

**Table 1:** Risk factors for CRC in IBD patients.

Risk factors	Disease duration > 8 years
	Disease extension
	Primary sclerosing cholangitis
	Family history of CRC
	Severity/persistence of inflammation
	Strictures
	Previous dysplasia
	Tubular colon
	Male sex
	Young age at UC diagnosis
	Post-inflammatory polyps

**Table 2:** Possible IBD drug-related malignancies.

Drug	Malignancy
Thiopurines	Lymphomas Myeloproliferative syndromes Non-melanoma skin cancers Urinary tract cancers Cervical carcinoma
Anti-TNF	Lymphoma Melanoma
Anti-TNF + thiopurines	Lymphoma
Vedolizumab	None identified
Ustekinumab	None identified
JAK inhibitors	Unknown
Methotrexate	Non-melanoma skin cancers

**Table 3:** Management of IBD patients with cancer history.

IBD Treatment Management
Withhold immunomodulation during active cancer therapy
Withhold thiopurines if bone marrow suppression is expected from the planned chemotherapy
Considerer avoiding immunomodulation in the first 2 years after cancer
Avoid combination therapy in cancer patients
Avoid JAK inhibitors in cancer patients

## Data Availability

Not applicable.
